# When biomolecules meet 2-hydrazinopyrazine: from theory through experiment to molecular levels using a wide spectrum of techniques†

**DOI:** 10.1039/d0ra06239a

**Published:** 2020-11-09

**Authors:** Paulina Mech, Mariusz Makowski, Anna Kawiak, Agnieszka Chylewska

**Affiliations:** University of Gdańsk, Faculty of Chemistry, Department of Bioinorganic Chemistry ul. Wita Stwosza 63 80-308 Gdańsk Poland agnieszka.chylewska@ug.edu.pl; Intercollegiate Faculty of Biotechnology of University of Gdańsk and Medical University of Gdańsk, Laboratory of Plant Protection and Biotechnology ul. Abrahama 58 80-307 Gdańsk Poland

## Abstract

The design of drug structures that are non-toxic, easily transported and permeable to cellular barriers is currently one of the most growing research trends. Indeed, the structural similarity of 2-hydrazinopyrazine (2HP) to pyrazinamide, which has been successfully used in anti-tuberculosis therapy, makes 2HP a promising research object. Thus, herein, a complete analysis of the structure of 2HP and its physicochemical and cytotoxic properties was performed. *Calf thymus* DNA (*CT*-DNA) and bovine serum albumin (BSA) binding studies were conducted, which demonstrated the higher affinity of 2HP to BSA. Furthermore, cytotoxicity tests were performed, which proved that 2HP was non-toxic to human skin keratinocyte cells. Accordingly, 2HP was initially classified as a compound with potential application. Physicochemical investigations were performed using a wide range of experiments, which were supported by DFT calculations using the B3LYP functional and 6-311+G** basis set. The good correlation, high quality and correctness of the obtained parameters were proven although the data was obtained using independent techniques. Additionally, 42 tautomeric (prototrophic) forms of 2HP were found by searching the conformational hyperspace. The most energy stable 2HP conformer structure and the partial charge distribution were established. The preferred 2HP ionic forms preferred were presented, and models of the equilibrium occurring in aqueous solution were proposed. The hydrophilic character of 2HP was established based on the partition coefficient values determined by both experiment and theory. The PCM and SMD solvent models of water and *n*-octanol were used.

## Abstract 2

Structure, ionic forms, hydrophilic and non-cytotoxic character of 2-hydrazinopyrazine with stronger affinity to BSA than DNA were proved.

## Graphical abstract

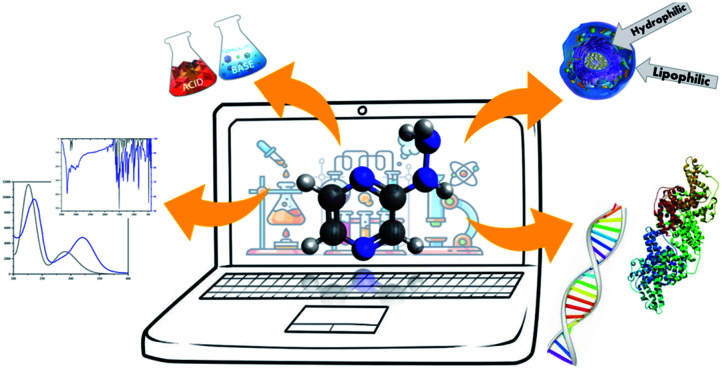

## Introduction

In recent years, compounds with aromatic and nitrogen-containing heterocyclic moieties represent the largest group of used medicines. Therefore, they are also very popular among scientists in the search for new compounds of biological importance or with possible pharmacological or environmental use.^1–3^ Thus, pyrazine derivatives fit well with the current research trend. Pyrazines contain an aromatic ring with nitrogen atoms (in the 1st and 4th positions of the six-member ring, respectively) and a substituent, commonly in the second position of the ring, which form a larger compound. The majority of the investigated pyrazine derivatives in the literature exhibit some biological activity,^4,5^ for instance herbicide, phytotoxic,^6,7^ anticancer,^8–10^ anti-inflammatory,^7,10,11^ antifungal,^6,7,12,13^ antioxidant,^14^ and antibacterial activity.^6,7,15–20^ Thus, their activity and simple structure make them an interesting group of compounds to perform physicochemical and biological studies.

Interestingly, one of the pyrazine family member is an anti-tuberculosis drug, pyrazinamide, a simple compound containing a pyrazine ring and an amide moiety.^21,22^ Its properties and activity have been known for a while, and thus currently, scientists are interested in its analogs, derivatives and complexes. Some of these compounds including pyrazine-2-aminoxime (PAOX), pyrazine-2-thiocarboxamide (PTCA), and 2-amine-5-bromo-3-(methylamine)pyrazine (ABMAP) were previously investigated by our group.^23–25^

In the discovery of biologically active compounds, there are several important issues such as structure, tautomerism, acid–base equilibrium, hydrophobicity, and membrane permeability. Knowledge of these properties allows the potential biological and therapeutic activities and behavior of compounds in the body or environment to be predicted.^26^ Accordingly, the aim of our studies was to determine the complete physicochemical characteristics of 2HP, including its structure, tautomerism, conformational isomerism, acid–base properties, lipophilicity, formation of intramolecular hydrogen bonds, DNA and protein binding ability, and cytotoxic activity.

An important aspect is the influence of tautomerism on the exact structure of a compound, and thus its potential interaction with biological systems. The majority of tautomeric equilibria in heteroaromatic compounds are prototrophic, which consist of proton migration with the simultaneous transfer of a double bond. Determination of the relative Gibbs free energies of tautomers and conformers allows their stability and structural participation to be assessed, and finally the form in which the compounds occur most often. The energy barrier of the transition between conformers is also an important factor, which allow the ease of transition from one form to another and the possibility of equilibrium to be determined.^27,28^

Acid–base properties are measured using the p*K*_a_ value (negative logarithm of an acid dissociation constant), is a very important parameter in specifying acidity or basicity in different media (solvent and environment). It is well known that proton transfer is one of the most fundamental and crucial reactions in nature, and thus the comprehension and prediction of its pathway and dissociation constants are significant. This physicochemical parameter is beneficial to define and comprehend the biological activity and behavior of active substances at the molecular level in the cell or biochemical processes (absorption, distribution, metabolism, and excretion), and their permeability, bioavailability and formulation.^29^ Moreover, the acid–base equilibrium influences molecular interactions and changes in compound charge with a change in pH. The p*K*_a_ values can be predicted using several methods, both experimental and theoretical. Potentiometric titration and UV-Vis spectrophotometry are the most commonly used experimental methods, and the theoretical methods are usually based on the thermodynamic cycle or isodesmic method.^30–34^

Lipophilicity (hydrophobicity) is related to the ability of a compound to cross the cell membrane or natural barriers at the cellular level. Membranes control the behavior of compounds, and therefore they take part in all relevant biochemical processes that determine the pharmacokinetics of the compound. The ability of a molecule to insert into biological membranes is regulated by its hydrophobicity, and thus hydrophobicity is the main factor in determining the compound distribution.^35^ Lipophilicity plays a key role in obtaining the solubility of a compound in an environment and clarification of its interactions with bioelements. It allows the possibility of action and durability of the compound in the natural environment to be determined. The lipophilicity of solutes is usually expressed as the partition coefficient (log *P*), which can be determined in a model system of two immiscible solvents, such as the octanol/water partition system. Water represents the main intracellular environment, whereas hydrophobic octanol is used due to its similar properties to the lipid bilayer. However, log *P* refers only to a neutral molecule.^36–38^ The partition coefficient can be measured experimentally in various ways, including the shake-flask, reverse phase HPLC, and pH-metric methods, or estimated by theoretical calculations.^39–41^

After solving the structure of scientific interest and determining its physicochemical properties, it is beneficial to investigate its binding properties using biomolecule assays. Accordingly, the possible binding ability of 2HP with DNA and bovine serum albumin (BSA) protein was investigated in detail experimentally. The interactions of small molecules known as drugs or ligands with DNA have been the subject of recent research in the field of life sciences, medical chemistry, biochemistry, and bioinorganic or coordination chemistry. These are particularly important studies in the context of the impact of a compound with possible biological use. It is important to know the possible interactions of compounds with both DNA and proteins to exclude negative impacts on living organisms.

2-Hydrazinopyrazine (2HP) is an analogue of pyrazinamide (PZA), where instead of the amide group, a hydrazine group is attached in the second position of the ring. However, is no description of its complete physicochemical properties in the literature. Our work, to the best of our knowledge, is the first complete analysis of the structure, lipophilicity, acid–base and cytotoxic properties of 2HP. Thus, for the most accurate and reliable study, we combined theoretical and experimental research. Considering that pyrazines have various biological activities, we also suspected that 2HP has similar activity. Therefore, studies were performed to determine its ability to bind to biomolecules and its cytotoxicity.

## Computational details

All quantum chemical calculations were performed using the Gaussian 09 (ref. 42) software package. The calculations for the conformational isomerism and tautomerism analyses were conducted in the gas phase using the DFT approach with the B3LYP (Becke's three parameter Lee–Yang–Parr) functional^43^ and the split-valence 6-311+G** basis set of Pople's group.^44^ The molecular geometries were fully optimized, and the total free energies were calculated at the same level of theory. The proton transfer process analysis was performed using the IRC procedure with the B3LYP/6-311+G** method level *in vacuo*.

Vibrational frequencies were also computed at the same level of theory and used to generate the IR spectrum of 2HP. The electronic absorption spectrum (UV-Vis) of the optimized 2HP molecule was calculated using time-dependent density functional theory (TD-DFT) at the B3LYP/6-311+G** level.

Acid–base properties were examined in water. All the gas-phase minimum geometries were optimized using the PCM^45,46^ and SMD^47^ solvation models at the B3LYP/6-311+G** level. The solvent is modelled as a polarizable continuum by these two methods. The p*K*_a_ values were determined based on the thermodynamic cycle proposed by J. A. Keith and E. A. Carter.^48^ It was assumed that the Gibbs free energy of a proton is −6.28 kcal mol^−1^*in vacuo* and −265.9 kcal mol^−1^ in water, and the correction for the change in the Gibbs free energy arising from the conversion of the standard states (1 M to 1 atm) is 1.89 kcal mol^−1^.^34,49^

Partition coefficient studies were performed in a water/*n*-octanol system. Gas phase minima geometries were optimized at the PCM/B3LYP/6-311+G** and SMD/M06-2X/6-311+G** levels in water and *n*-octanol. The Gibbs free energies obtained as a result of these calculations in water and *n*-octanol were used to determine the log *P* value.^41^ Details can be found in ref. 23.

All calculations were executed at a temperature of 298.15 K and pressure 1 atm or standard state 1 M in the gas phase and solvent model, respectively.

## Experimental

### Materials

The main 2-hydrazinopyrazine (2HP) material and the other chemicals used during the experimental investigations, *e.g. n*-octanol (anhydrous, ≥99%), KOH (solid and eluent concentrate 0.1 M, aq.), HCl (37%, aq. ACS reagent), HClO_4_ (70%, aq. ACS reagent) and sodium perchlorate monohydrate, NaClO_4_·H_2_O (puriss., ACS reagent, ≥98.0%) were obtained from Sigma-Aldrich. The pH-meter calibration buffers were obtained from Mettler Toledo GmbH, Germany. All solutions were prepared directly before the experiments using Hydrolab-Reference purified water with conductivity not exceeding 3.0 μS cm^−1^.

### Potentiometry procedures and equipment

The titration system consisted of a titration cell, magnetic stirrer and automatic microtitrator with a Hamilton syringe with the volume of 0.5 mL and step of volume 0.004. 9.8 mM KOH (carbon dioxide free) solution was used as the titrant. Potentiometric titrations were performed on a CERKO LabSystem automatic titrator applying the CERKO program, using InLab 423 combined glass–Ag/AgCl electrodes (Mettler Toledo). The combined electrode was used after calibration of its parameters (*E*^0^ = 382.16; *S* = −54.06) using freshly prepared solutions. The temperature of the measurement was maintained at 25 ± 0.1 °C. The samples containing 2HP (0.31 mM) were dissolved in HCl (0.99 mM). The ionic strength of all the working solutions was controlled using NaClO_4_ (10.01 mM). The sample volume of 2.5 mL with the abovementioned composition was used to perform each potentiometric titration. The experimental potentiometry data was analyzed using the CVEQUID program,^50^ which is based on an algorithm that matches the assumed equilibrium model to measurement data, and the iterative method of Gauss–Newton–Marquardt^51^ was used to solve nonlinear problems. All experiments were performed in the pH range of 3.12 to 11.88. The resolution of the voltage measurements was <0.1 mV. The titrations were performed in triplicate and used to calculate the deprotonation of the studied 2HP solution.

### Spectrophotometric investigations

All the absorption spectra were recorded with a Thermo Scientific Evolution 300 spectrophotometer in the wavelength range of 200–500 nm and 200–400 nm for the spectrophotometric and Hammett UV microtitrations, respectively. All titration solutions were maintained at a constant temperature of 25.0 °C (±0.1) by circulating thermostated water through the spectrophotometer cuvette holders, and for the external titrations, through the external titration vessel. The stability constants of the complexes were determined using the EQUID software^52^ by minimizing the differences between the theoretical model and the experimental data.

^1^H NMR spectra were recorded with a Bruker AVANCE 700 MHz spectrometer at the NMR Laboratory at the Faculty of Chemistry (University of Gdańsk). The chemical shifts in the proton NMR spectra were obtained using ten 2HP samples in a mixture of H_2_O/D_2_O (9 : 1 = v/v) as the solvent at selected pH of 2.41, 5.16 and 11.68. The ionic strength of the solutions was kept constant using 0.1 M NaClO_4_. The additional acidified sample was prepared at the required pH in the mentioned mixture with the addition of 70% HClO_4_ (*H*_o_: −0.81; the ionic strength was maintained intentionally using 0.3 M NaClO_4_ for this probe only). The presented pH values are the measured values of the mixture in the presence of 2HP. NMR measurements were performed at 298 K.

### Partition (*P*) coefficient experimental details

Initially, the calibration curves for 2HP in both solvents studied, octanol and water, were measured. Briefly, a freshly prepared sample of 2HP in *n*-octanol was carefully diluted with pure solvent using pipettes, test tubes and graduated cylinders to obtain a series of different, but known concentrations to create a calibration curve. The same procedure was repeated for 2HP dissolved in water, as presented in the ESI.† In the second step of the experiment, a stock solution of 2-hydrazinopyrazine with a concentration of 1.11 × 10^−4^ M (50 mL) was prepared in octanol. Stock octanolic solutions (5 mL) of 2HP were assigned as probes (1)–(5). The corresponding volumes of pure water (5 mL) were added to each of them and the extraction process was started individually. A ring stand was set up to hold a separatory funnel, in which the stock solutions were poured followed by water. The funnel was then removed from the stand and strongly shaken for about 30 min with intermediate venting. It was then left to rest in place on the ring stand to allow the substances to equilibrate into two phases. Once distinct layers appeared after about 45 min, equilibrium was assumed to have been reached. Five independent 2HP extractions in the OCT/W systems were carried out for the determination of the partition and distribution coefficients, and the experimental conditions and the graphical forms of the results are presented in the ESI.† Samples (1)–(5) of 2HP after extraction were grouped as octanolic (1)_OCT_–(5)_OCT_ and aqueous (1)_W_–(5)_W_ fractions, respectively, and some solution of each sample was pipetted into a cuvette to record its spectrum.

### Affinity to biomolecule assay

The UV-Vis titration assay was carried out in tris-(hydroxymethyl)-amino methane (Tris–HCl) buffer solution (5 mM Tris–HCl, 50 mM NaCl, pH 7.39). A solution of *CT*-DNA (Sigma-Aldrich) in Tris–HCl gave the UV-Vis absorbance ratio of 1.8–1.9 at 260 and 280 nm as the standard procedure to indicate that DNA was sufficiently free of protein. The titrations were performed automatically using a CerkoLab microinjector at 25 °C in the wavelength range of 200–400 nm. Electronic absorption spectra were recorded after the addition of different amounts of individual biomolecule solutions.

The concentration of the freshly prepared *CT*-DNA was calculated based on the absorbance value at 260 nm and the calibration curve [*ε*_DNA_ 6600 (base pairs) per M per cm].^53^ 2HP was dissolved in Tris–HCl to give 10 mL of solution at a mass concentration equal to 0.18 mM, which was used as the initial and pure pyrazine derivative sample for both types of titration.

1.5 mL solution containing the appropriate concentration of 2HP was titrated by the successive addition of a 0.25 mM stock solution of bovine serum albumin (BSA). The concentration of the freshly prepared BSA (titrant solution) was established based on the calibration curve obtained at 278 nm.

### Cell culture and cytotoxicity assay

The human skin keratinocyte cell line HaCaT (Cell Line Services, Germany) was cultured in high-glucose Dulbecco's Modified Eagle Medium (Sigma-Aldrich, Germany) supplemented with 10% fetal bovine serum (Sigma-Aldrich, Germany) and 100 μg mL^−1^ streptomycin and 100 units per mL penicillin (Sigma-Aldrich, Germany). The cultures were maintained in a humidified atmosphere at 37 °C with 5% CO_2_. Cell viability was assessed using the MTT (3-(4,5-dimethylthiazol-2-yl)-2,5-diphenyltetrazolium bromide) assay. 2-Hydrazinopyrazine was analyzed in the concentration range of 1–100 μM. The MTT assay was performed by seeding cells (5 × 10^3^ cells per well) in 96-well plates and treating them for 72 h with the examined compound. Subsequently, MTT (0.5 mg mL^−1^) was added to the wells and incubated for 3 h at 37 °C, after which the medium was discarded, and the formed formazan crystals were dissolved with DMSO (100 μL per well). The optical density of the formazan solution was measured at 550 nm using a plate reader (Victor, 1420 multilabel counter).

### Statistical analysis

Values are expressed as mean ± SE of three independent experiments. Statistical analysis was performed using GraphPad Prism 5.0 (GraphPad software). Differences between the control and 2-hydrazinopyrazine-treated samples were analyzed by one-way ANOVA with Tukey's *post hoc* tests. A *p* value of <0.05 was considered statistically significant in each experiment.

## Results and discussion

### Structure

**Fig. 1:**
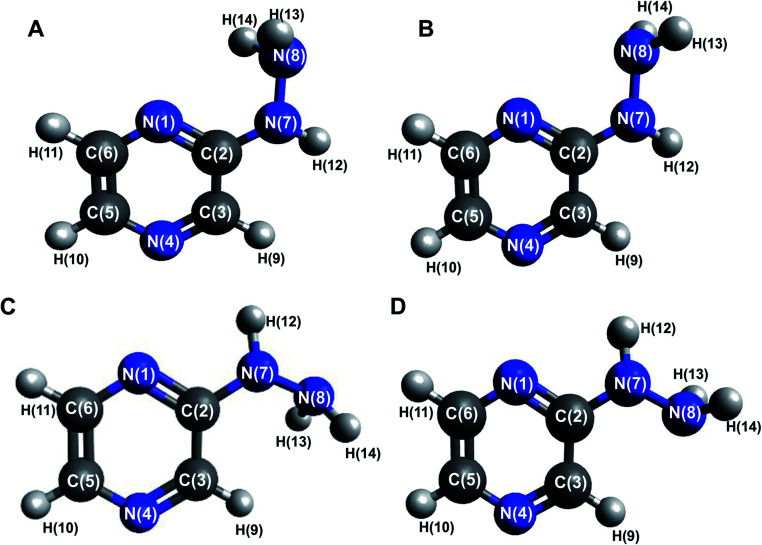
Optimized conformer structures of 2-hydrazinopyrazine (2HP) with atom numbering. Their relative Gibbs free energies in kcal mol^−1^: (A) Δ*G* = 0.00, (B) Δ*G* = 4.98, (C) Δ*G* = 1.16 and (D) Δ*G* = 0.82.

The 2HP molecule with the pyrazine ring substituted in the second position with a hydrazine group was characterized in the minimum of the potential energy surface by a planar ring, with the nitrogen framework of the substituent being almost co-planar with the ring. The optimized geometries with atom numbering are shown in Fig. 1. The minimum Gibbs free energy obtained from the structure optimization of 2HP is −375,028923 a.u. The bond lengths, angles and selected dihedral angles of this optimized structure are listed in Table S1 of the ESI.† The bond lengths in the aromatic pyrazine ring are in the range of 1.319 to 1.418 Å and that for all the bonds with hydrogen atoms is in the range of 1.008 to 1.088 Å. The C2–N7 bond has a length of 1.379 Å and the N7–N8 bond has a length of 1.408 Å. The angles within the ring were measured to be between 116.447° and 122.568°. The angle of N1–C2–N7 is 118.551°. Slight deformations in the ring (especially N1–C2–C3) relative to pyrazine can be observed,^54^ which are caused by the presence of a hydrazine group attached at the second position of the ring.

The distribution of partial charges is directly related to the vibrational properties of the molecule and its chemical bonds. It affects the dipole moment, polarizability, electronic structure and other properties of molecular systems. Using the NBO method, the partial charges of 2HP were determined, and the results are presented in Table S2 of the ESI.† The results show that the carbon atoms connected with hydrogen atoms have negative charges except for C2. The positive C2 charge results from the effect of electron extraction by two associated nitrogen atoms. All the nitrogen atoms have negative charges, and the minimum value is located on the N8 atom (−0.80414). Consequently, all the hydrogen atoms have a positive charge. The atoms bound with nitrogen atoms have a higher charge (about 0.26) than that associated with carbon atoms (about 0.42).

### Frontier molecular orbitals (FMOs)

**Fig. 2:**
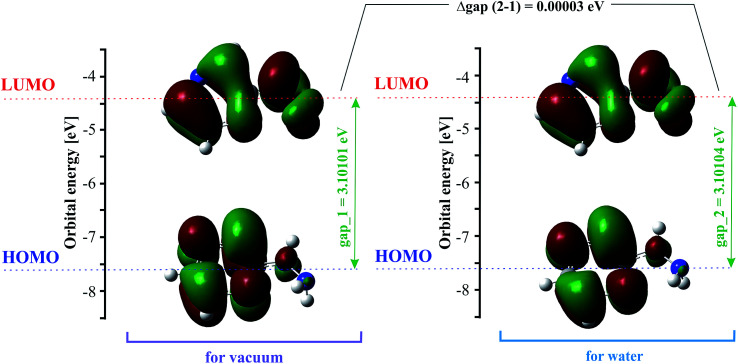
HOMO and LUMO for 2HP at the B3LYP/6-311+G** level.

The highest occupied molecular orbital (HOMO) and the lowest unoccupied molecular orbital (LUMO) are also named frontier molecular orbitals (FMOs). The HOMO is related to the ability to donate electrons, and the LUMO as an electron acceptor is associated with the ability to attach an electron. The energy gap between the HOMO and LUMO (difference between the HOMO and LUMO energy) determines the chemical stability and reactivity, kinetic stability, optical polarizability and chemical hardness–softness of a molecule.^55,56^ The HOMO and LUMO distribution with the energy gap is shown in Fig. 2. The negative and positive phases are marked in green and red, respectively. The HOMO orbital is located over the pyrazine ring, whereas the LUMO orbital is spread over the entire molecule, *i.e.* both the hydrazine group and pyrazine ring. The HOMO → LUMO transition involves an electron density transfer from the pyrazine ring to the hydrazine group. The calculated energy values of the HOMO are −7.57782 and −7.57789 eV, and LUMO are −4.47681 and −4.47685 eV in the gas phase and water, respectively. The HOMO–LUMO gap energy is 3.10101 eV *in vacuo* and 3.10104 eV in water. This quite large energy gap indicates the high stability of 2HP.

### Vibrational and UV-Vis spectral analysis

**Fig. 3:**
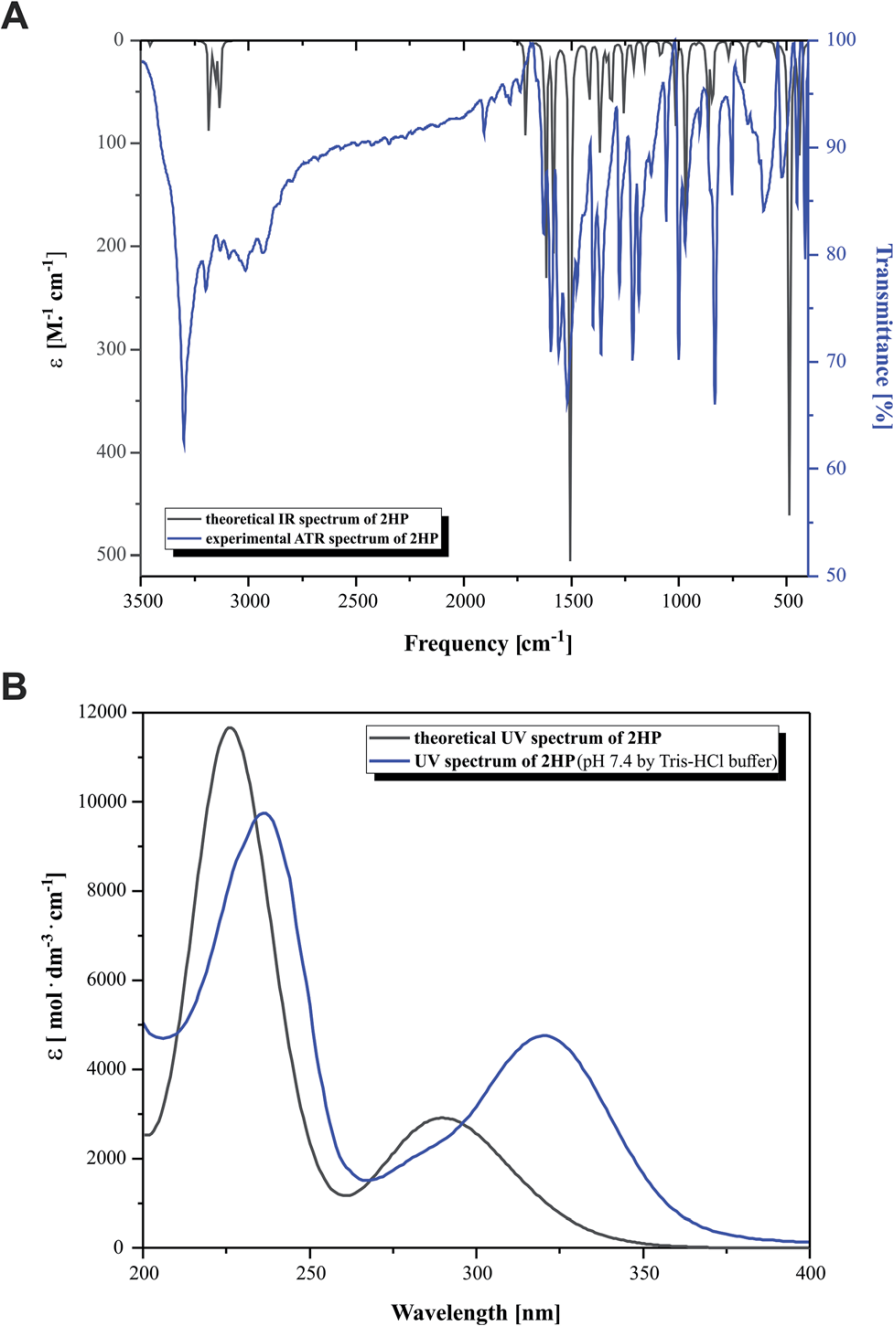
(A) Simulated (black) and experimental (blue) IR spectra and (B) simulated (black) and experimental (blue) UV-Vis spectra.

The experimental and the theoretical vibrational spectra of 2HP were plotted as intensity *versus* vibrational frequency, as shown in Fig. 3. The theoretically predicted vibration spectrum has no imaginary wavenumber, which means that the optimized geometry on the surface of the potential energy is at the local lowest point. The calculations were performed for a single molecule *in vacuo*, while the experiments were carried out using solid samples; therefore, there are inconsistencies between the calculated and observed vibrational wavenumbers and intensity. The wavelength values obtained theoretically and experimentally are consistent in quality, but not necessarily as numerical values, which is also due to the approximate nature of theoretical methods.

Heteroaromatic compounds with an amine group show N–H stretching vibrations in the range of 3500–3220 cm^−1^. In the spectrum of 2HP, the bands at 3456 cm^−1^ and 3528 cm^−1^ were assigned to the symmetric and asymmetric vibration modes of the NH_2_ group, respectively. The C–H vibrations appeared in the range of 3000–3100 cm^−1^, which is the characteristic region for C–H vibrations and may indicate a heteroaromatic structure. The bands located at 3232, 3155 and 3182 cm^−1^ in the spectrum of 2HP can be attributed to the C–H stretching vibrations. For six-membered aromatic rings, *e.g.* benzene and pyridines, there are two or three bands in this region due to their skeletal vibrations, with the strongest usually located at about 1500 cm^−1^. The bands observed at 1503 and 1508 cm^−1^ are assigned to the C–C ring vibrations. For monosubstituted pyrazines, very strong, strong and weak bands are observed in the region 1025–1000, 840–785 and 660–615 cm^−1^, respectively. In this study, the bands identified at 1159, 1084, 1015 and 627 cm^−1^ in the IR spectra are attributed to the C–H ring in-plane bending modes. The C–N in-ring bending vibrations were identified at 1582 and 1418 cm^−1^. Additionally, the peaks located at 968, 865 and 844 cm^−1^ are ascribed to the C–H out-of-plane vibrations. The bands located in the range of 1253 to 1365 cm^−1^ are attributed to the C–N and N–H stretching vibration modes. The N–H scissoring vibration is usually observed in the range of 1650–1620 cm^−1^; however, in this study it was observed at 1617 cm^−1^.^57–60^

Fig. 3B presents the experimental UV-Vis (blue) and theoretical gas phase (black) spectra, which were recorded in the wavelength range of 200–400 nm. Based on the fully optimized ground-state structure, TD-DFT/B3LYP/6-311+G** calculations were performed to determine the low-lying excited states of 2HP. The calculated absorption maxima were found to be located at 226 and 291 nm in the gas phase and at 236 and 320 nm in the experimental spectra, which are ascribed to the π → π* and n → π* transitions, respectively.

### Conformational isomerism

Four conformers were found upon searching the conformational hypersurface. Calculations on each conformer were performed to determine their relative stability *in vacuo*, and their optimized geometries with atom numbering and relative Gibbs free energies in kcal mol^−1^ are presented in Fig. 1. The DFT calculations showed that the lowest-energy, and thus the most stable conformer, is 2HP(A). Its pyrazine ring is a flat system connected to the hydrazine group with a single bond C2–N7, which is a place of possible rotation. Conformers 2HP(A) and 2HP(C) are obtained by rotation around the C2–N7 single bond. Consequently, conformers 2HP(B) and 2HP(D) are obtained from 2HP(A) and 2HP(C) through rotation around the N7–N8 single bond. Conformational structures are usually in a state of dynamic equilibrium, which means that they are continuously changing into each other, which is confirmed by the small energy differences between the conformers. In these systems, some of the structures (with the lowest energy) are energetically privileged, and thus their share is greater than for the other structures. In the case of the 2HP conformers (B-D), the Gibbs free energy differences are in the range of 0.82–4.98 kcal mol^−1^ compared to the lowest energy conformer (A), indicating their significant participation in the 2HP structure.

**Fig. 4:**
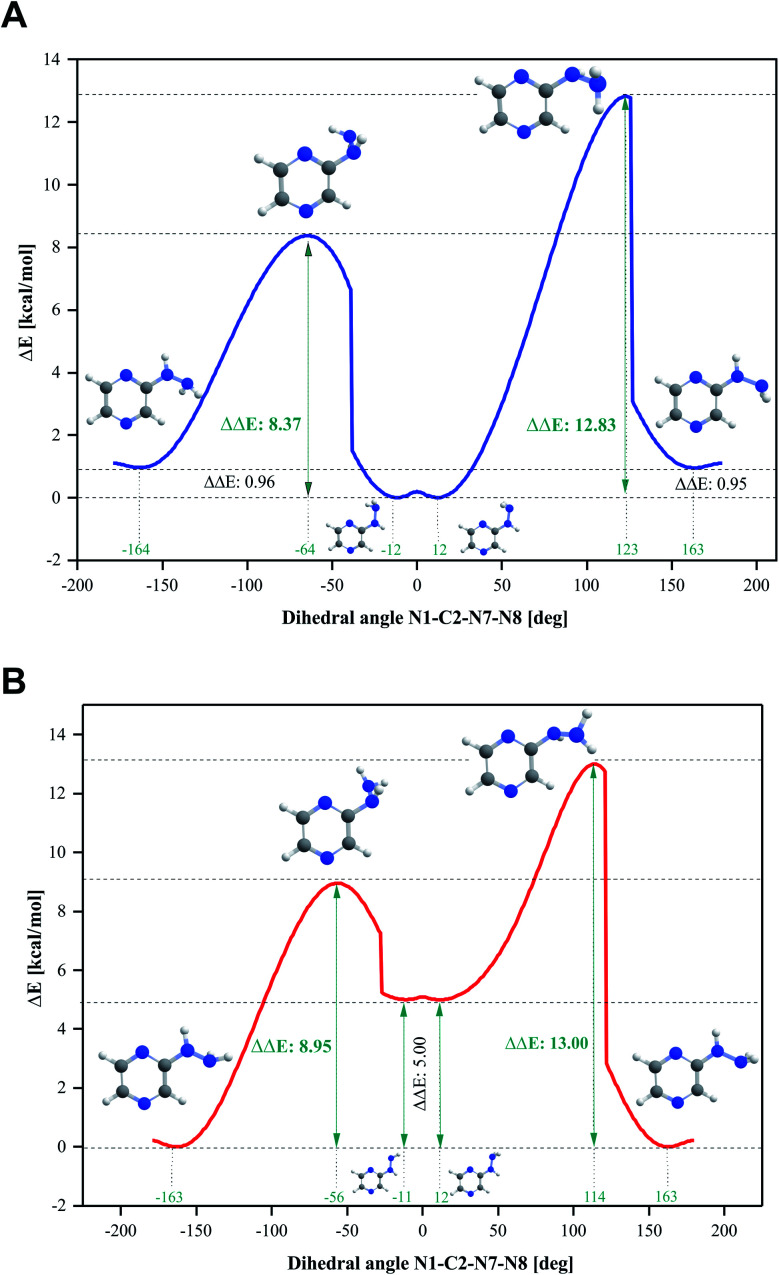
Energy variation curve of the dihedral angle of N1–C2–N7–N8 in 2-hydrazinopyrazine during the transition of the conformers. (A) 2HP(A) into 2HP(C) and (B) 2HP(B) into 2HP(D).

The calculation results clearly indicate that the most stable conformer is 2HP(A), and three other stable forms were also found. Scans of the C2–N7 bond rotation during the transitions of conformer A into C and B into D were conducted. The energy variation curve for the N1–C2–N7–N8 dihedral angle is shown in Fig. 4. The curve was drawn based on a series of energy minimizations with the integration step of 1° (at each value of the N1–C2–N7–N8 dihedral angle, constrains were imposed), and the remaining degrees of freedom were optimized. The graph obtained during the transition from 2HP(A) to 2HP(C) (Fig. 4A) shows four minima, corresponding to the dihedral angles of −12° and 12° with the energy as a global minimum, and −164° and 163° with the relative energy 0.96 and 0.95 kcal mol^−1^, respectively. The energy difference between the respective pairs of minima is 0.95/0.96 kcal mol^−1^, which is similar to the difference between conformers A and C. The second (−12°) minimum refers to conformer A, and the first (−164°) minimum refers to conformer C. The maxima are located at the dihedral angles of −64° and 123°, and the energy barriers between minima are 8.37 and 12.83 kcal mol^−1^, respectively.

The transition from 2HP(B) to 2HP(D) (Fig. 4B) is qualitatively similar to that in Fig. 4A. The graph shows four minima at dihedral angles of −163° and 163° with energy as a global minimum, and −11° and 12° with the relative energy 5.00 kcal mol^−1^ for both, respectively. The energy difference between the minima is similar to the difference between conformers B and D (Fig. 1). The second (−11°) minimum refers to conformer B, and the first (−164°) minimum refers to conformer D. The maxima are located at the dihedral angle −64° and 123°, and the energy barriers between minima are 8.37 and 12.83 kcal mol^−1^, respectively.

### Tautomerism (prototrophy)

**Fig. 5:**
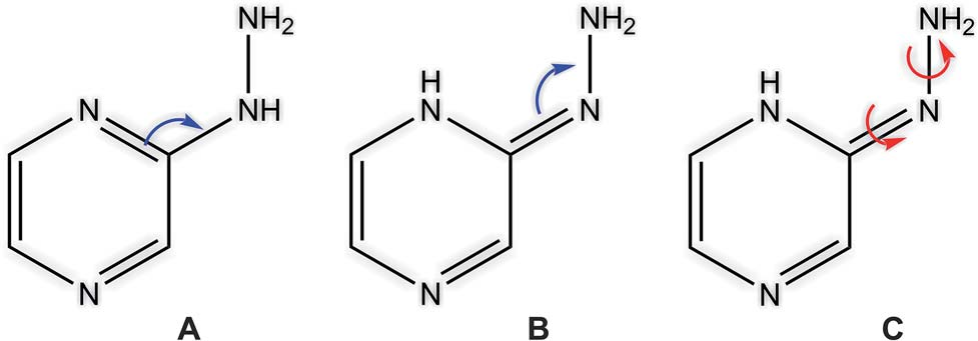
Considered changes in the position of the double bond (A and B) and rotation around the C2–N7 and N7–N8 bonds in the prototrophic forms (C).

Among heterocyclic compounds, the common type of tautomerism observed is prototrophy. This phenomenon involves the relocation of a hydrogen atom within a molecule together with a double bond transfer. 2-Hydrazinopyrazine has several protons that can be transferred this way. Fig. 5 schematically presents the considered changes in the position of the double bond (Fig. 5A and B) and additional rotation around the N7–N8 bond (Fig. 5C) in all the observed prototrophic forms. A total of 42 tautomeric forms were found, among which 12 are prototrophic forms of 2HP(A) and the remaining 30 are their conformers obtained by rotation around the N7–N8 single bond. Their optimized molecular structures and relative Gibbs free energies are presented in Table S3.† The relative Gibbs free energies of 39 of the 42 observed forms are in the rang of 20.83–47.06 kcal mol^−1^ compared to 2HP(A). These large energy differences indicate a small percentage of these structures exists, and thus finding them using experimental methods is not possible. Only three of them have relative Gibbs energies less than 15 kcal mol^−1^, and thus they can exist and participate in the structure, which are the PA, PA1 and PB tautomers presented in Table S3 of the ESI.†

### Proton transfer process

An important aspect in the analysis of the structure and energy stability of a compound is the possibility of the formation of intramolecular hydrogen bonds. The 2HP hydrazine group contains hydrogen atoms at the N7 and N8 nitrogen atoms, which can be hydrogen bond donors, while in the pyrazine ring, the strongly electronegative nitrogen N1 can be a hydrogen bond acceptor. Considering the relative position of the hydrazine group and the pyrazine ring relative to each other, an intramolecular hydrogen bond may occur in conformer 2HP(A) between the N8–H14⋯N1 atoms, and in the 2HP(C) and 2HP(D) conformers, between N7–H12⋯N1 (same arrangement). The proposed bonds and their lengths are shown in Fig. S1 included in the ESI.† In addition, an analysis of the proton transfer involved in the formation of a hydrogen bond along these bonds was carried out in the case of conformers A and D (conformers with the lowest relative Gibbs free energies). The total energy variation curves on the IRC path length during the IRC proton transfer process analysis are shown in Fig. S2 in the ESI.† The differences between the substrate and product in the proton transfer process for 2HP(A) and 2HP(D) are 31.07 and 13.81 kcal mol^−1^, respectively. The energy barriers of the proton transfer for 2HP(A) and 2HP(D) are 35.86 and 49.00 kcal mol^−1^, respectively.

### Acid–base properties

**Fig. 6:**
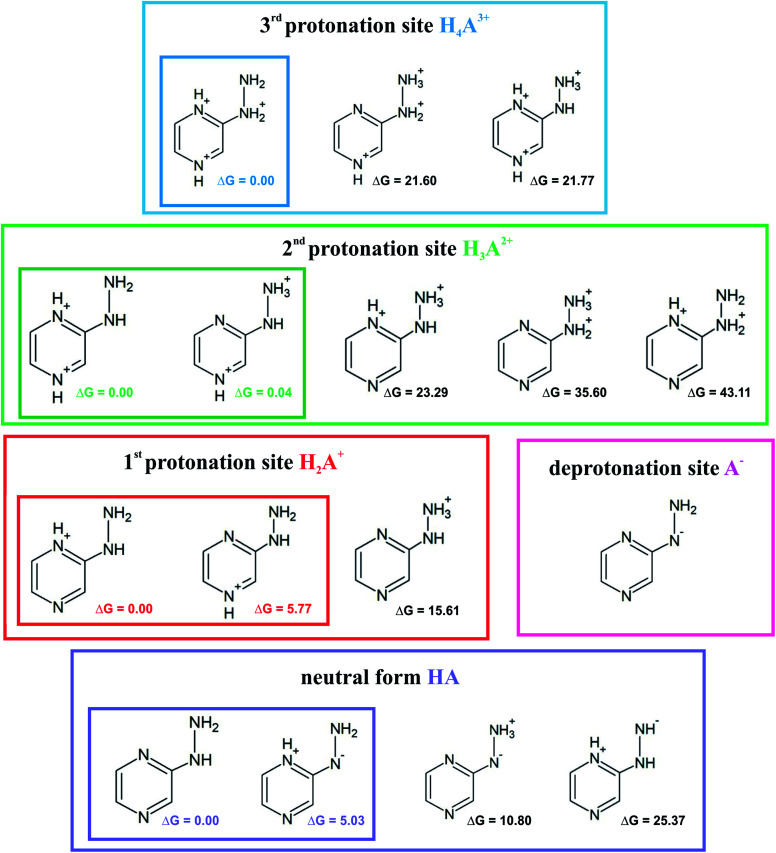
Proposed ionic forms present in the solvent together with the relative Gibbs free energies (Δ*G*) in kcal mol^−1^ for each protonation site and neutral (zwitterion) form.

Investigation of the acid–base properties began with the quest to determine the ionized forms of 2HP in the gas phase. To find the preferred ionized forms, the possible input structures were created and optimized. 2-Hydrazinopyrazine contains more than one atom that can be protonated, which are the nitrogen-heteroatoms in this environment. Three possible protonation sites and one deprotonation site were found. The optimized molecular structures and their relative Gibbs free energies are shown in Fig. 6.

The sites with the lowest relative energies are marked in Fig. 6, which are most likely the states found in solution and involved in the acid–base equilibrium. An aqueous medium was chosen to determine the acid–base properties because it is the environment most biochemical reactions occur and represents the conditions inside living cells. Based on the energy analysis, a model of the equilibrium occurring in aqueous solution was proposed. The p*K*_a_ values were determined (Table 1) by a direct method, which involves the following proton removal reactions:

1 BH_(aq)_^+^ ⇄ B_(aq)_ + H_(aq)_^+^

2 HA_(aq)_ ⇄ A_(aq)_^−^ + H_(aq)_^+^

**Table:** Proposed equilibria in water system based on theoretical calculations with regards to the forms shown in Fig. 6

Conjugated ionic forms of 2HP involved in equilibrium	Assumed model of direct reaction	Deprotonation constant (acidity)
Trication/dication	H_4_A^3+^ ⇆ H_3_A^2+^ + H^+^	p*K*_a_1__
Dication/monocation	H_3_A^2+^ ⇆ H_2_A^+^ + H^+^	p*K*_a_2__
Monocation/neutral	H_2_A^+^ ⇆ HA + H^+^	p*K*_a_3__
Neutral/monoanion	HA ⇆ A^−^ + H^+^	p*K*_a_4__

The calculations were based on a known thermodynamic cycle, which combines the gas-phase deprotonation Gibbs free energies and the solvation energies described elsewhere^34,48^ by the expression:3
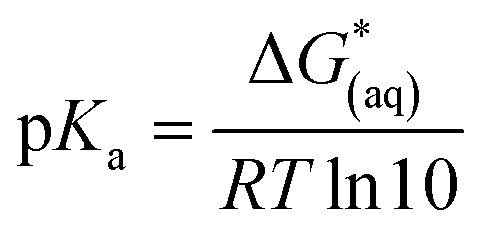
where 
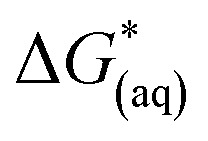
 is the Gibbs free energy change at standard state of 1 M.

The DFT simulations and experimental studies confirmed the existence of four acid–base equilibria. The calculated and theoretical p*K*_a_ values are shown in Table 2. The acid dissociation constants from the DFT calculations were obtained in relation to the proposed equilibrium in the water system presented in Table 1. The calculated values (Table 2B) obtained at both levels of theory are relatively compatible with each other. The two negative values can be associated with the occurrence of acid–base equilibrium within the pyrazine ring, and two positive values referring to the hydrazine substituent. There is also a consistent changing trend for the values from both the theoretical and experimental results. Specifically, they are qualitatively consistent, but vary quantitatively. This may be due to the approximations used in the theoretical calculations with the solvent models. The main source of errors in the p*K*_a_ calculations is relevant to the change in the Gibbs free energy of solvation, which was estimated using implicit solvation models. There is no perfect model for predicting constant values that would allow values fully consistent with the experiment to be obtained.

**Table:** The dissociation constant values obtained experimentally for (A) protonated 2HP determined at 25 °C in aqueous medium from *n* titrations (*I* = 10.01 M NaClO_4_; *n* ≥ 3); standard deviations computed thereby refer to random errors only. (B) p*K*_a_ values obtained by DFT approach

Method	p*K*_a_ value			
	p*K*_a_1__	p*K*_a_2__	p*K*_a_3__	p*K*_a_4__
**(A) Experimental part**				
Potentiometric titration	—	—	4.44 ± 0.08	10.56 ± 0.02
Spectroscopic titration	—	—	4.50 ± 0.09	10.96 ± 0.11
Spectroscopy Hammett	−3.44 ± 0.02	−1.06 ± 0.04	—	—

**(B) Theoretical part**				
PCM/B3LYP/6-311+G**	−32.12	−14.76	4.21	16.99
SMD/M06-2X/6-311+G**	−31.36	−10.48	6.32	13.12

### Spectroscopic and potentiometric results

Initially, the acid–base behavior of 2HP was considered because this knowledge allows their ionic forms to be predicted, which will be present in body fluids. Additionally, the p*K*_a_ value makes it possible to determine the solubility of a compound, *e.g.* (pro)drug or pharmaceutical. This enables the proper selection of dosage of a compound that produces the expected therapeutic, diagnostic or microbiological effects. The structures of compounds or, more precisely, the presence of donor functional groups, have a key influence on their biological and pharmacological activity. It is worth noting that many parameters used to profile drugs are directly related to their p*K*_a_ values, *e.g.* the degree of penetration in biological barrier. Moreover, the current research, such as Liberation Absorption Distribution Metabolism Excretion (LADME), to determine the fate of a given drug/pharmaceutical in the human body and their active substances always consider its p*K*_a_ values. These types of studies are carried out in both the initial and advanced clinical research steps.

**Fig. 7:**
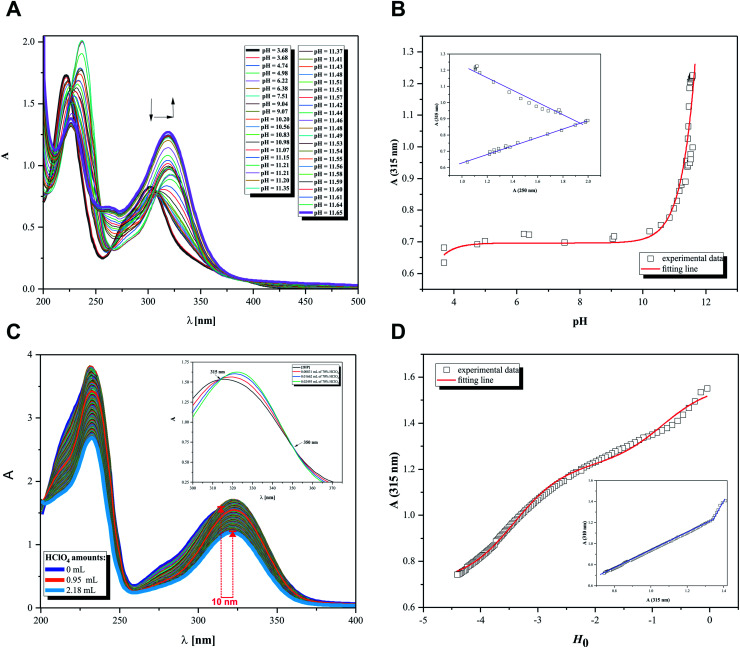
Spectrophotometric results. (A) UV spectral curves of 2HP (0.18 mM) as a function of pH (3.68–11.65). (B) Absorption at 315 nm *vs.* measured pH, where experimental data presented as squares and calculation results shown as red line; *R*^2^ = 0.98721. (C) Titration curves from Hammett's acidity function. (D) Absorption at 315 nm *vs. H*_o_ index, where experimental data presented as squares and calculation results as red line; *R*^2^ = 0.99647.

**Fig. 8:**
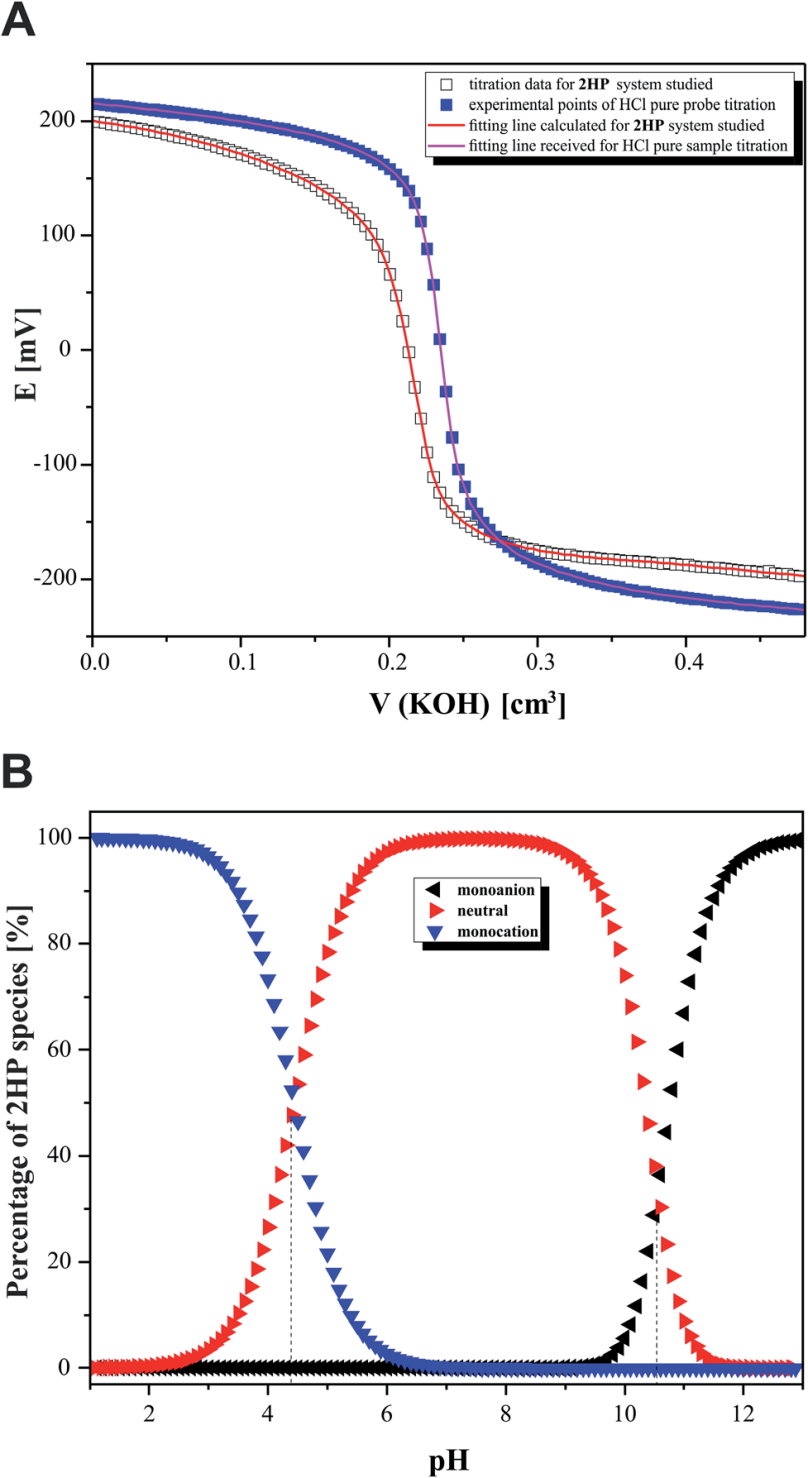
Potentiometric titration data. (A) Experimental points (black squares) for the potential and KOH (9.8 mM) volume relationship for 0.31 mM 2HP dissolved in 0.99 mM HCl/10.01 mM NaClO_4_ together with the result of CVEQUID calculations shown as a red line, where the plot *E* = *f*[*V*(KOH)] experimentally obtained (blue circles) refers to 2.5 mL of HCl pure probe (0.99 mM) with NaClO_4_ (10.01 mM) and titrated by KOH (9.8 mM) together with the green fitting line generated by CVEQUID. (B) Concentration diagrams for individual 2HP ionization states formed in the aqueous system studied.

Thus, pH-dependent spectrophotometric (Fig. 7) and potentiometric titrations (Fig. 8) were carried out to characterize the behavior of 2HP in an aqueous environment. Microtitration experiments for the determination of the complete acid–base properties of 2HP based on a variety of measurable parameters were also planned and developed with the aim to support the theoretical results. Initially, UV spectroscopic studies of 2HP with respect to a change in pH were performed, and the electronic spectra were recorded. The low wavelength part of the spectra was dominated by very broad and intense bands, originating from the double bonds of the pyrazine scaffold present in the 2HP structure. It can be seen that the first deprotonation process of the 2HP system studied occurs spontaneously. For clarity, the initial spectrum of 2HP solution at pH 3.68 is presented in bold (black) in Fig. 7A, but the proton transfer of this process is related to the cationic form of 2HP according to the initial acidification of the 2HP parent sample. The intensity of the absorption band at 225 nm decreased, whereas the intensity of the band at 315 nm increased upon the addition of the titrant KOH in comparison to the final titration spectrum in bold registered at pH 11.65 (Fig. 7A). These spectral changes were accompanied by one isosbestic point (at 264 nm), which appeared at high pH values (above 11) and was present until the end of the titration. This observation suggests that equilibrium related with proton transfer may occur in this pH region. The exact number of equilibria occurring in the investigated system was obtained by plotting *A-*diagrams.^61^ The corresponding *A*-diagrams are presented in Fig. 7B, which show two linear sections for the various wavelength combinations. The spectroscopic 2HP pH-dependency studies and UV results proved that two equilibria occurred in the 2HP aqueous system studied. It is worth noting that the first step of 2HP deprotonation is directly associated with its monocationic state as a result of 2HP pattern sample acidification. The standard potentiometric methodology used here is aimed at widening the pH measurement range. Additionally, dissociation process models were proposed and created individually for the monoprotonated 2HP state experiments and 2HP tautomerization processes, which were considered in parallel as scientific objectives of the theoretical investigation.

The relationship between the absorbance wavelength at 315 nm and pH value was plotted, as shown in Fig. 7B. The p*K*_a_ constants were calculated using the standard procedure based on the Henderson–Hasselbalch equation.^23,62^

Additionally, spectroscopic measurements using Hammett's acidity function were also performed for 2HP. This investigation was planned based on the knowledge acquired from the physicochemical properties of other pyrazine derivatives reported by us previously.^23,24,62^ The initial step of the investigation included the preliminary determination of the relation between the HClO_4_ molar concentration and the *H*_o_ index. The obtained for 2HP results presented in Fig. 7C and D clearly show that two equilibria occurred during the titration of 2HP using 70% HClO_4_ (the *A*-diagram included in the inset of Fig. 7D shows two linear sectors). The 2HP protonation constant values for this measurement are related with the N heterocyclic atoms of the 2HP pyrazine ring. The p*K*_a_ values assigned to the protonation of the N atoms of the pyrazine ring were determined based on the equation converted by Hammett function (4) below and presented in Table 3.4
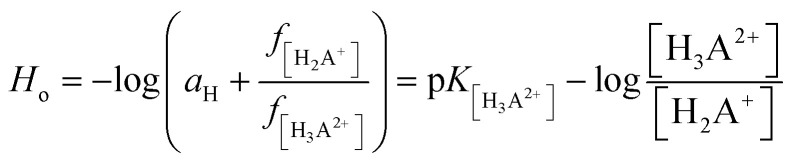


**Table:** Proposed deprotonation equilibrium models used for the aqueous systems studied in CVEQUID calculations (potentiometry and spectrophotometry), and 2HP neutral form was assigned as [HA]

Conjugated ionic forms of 2HP involved in equilibrium	Assumed model of protolytic reaction	Deprotonation constant (acidity)
Monocation/neutral	H_2_A^+^ + OH^−^ ⇆ HA + H_2_O	p*K*_a_1__
Neutral/monoanion	HA + OH^−^ ⇆ A^−^ + H_2_O	p*K*_a_2__
Water formation	H_3_O^+^ + OH^−^ ⇆ 2H_2_O	p*K*_aq_

In the second step of the investigation, potentiometric microtitrations (Fig. 8A) were performed to verify the p*K*_a_ values obtained from spectrophotometry. The potentiometric curves also confirmed that two equilibrium reactions of 2HP ionic species spontaneously occur in the pH range of 3.12–11.88. Interestingly, the potentiometric experiment allowed the percentage of 2HP individual species to be determined (Fig. 8B).

The data obtained from the calculation using the model created (Table 3) in the CVEQUID program^63^ is presented as adequate dissociation constant values (p*K*_a_) of 2HP and collected in the Table 2 together with the other experimental and theoretical effects. The analysis of the p*K*_a_ values suggests that the data was determined with high accuracy (see low errors given in Table 2). Moreover, the results correlate well although they were obtained through independent measurements, both spectrophotometric and potentiometric, with optimal but different conditions and different measurable parameters (absorbance and/or potential, respectively) of the studied 2HP system.

**Fig. 9:**
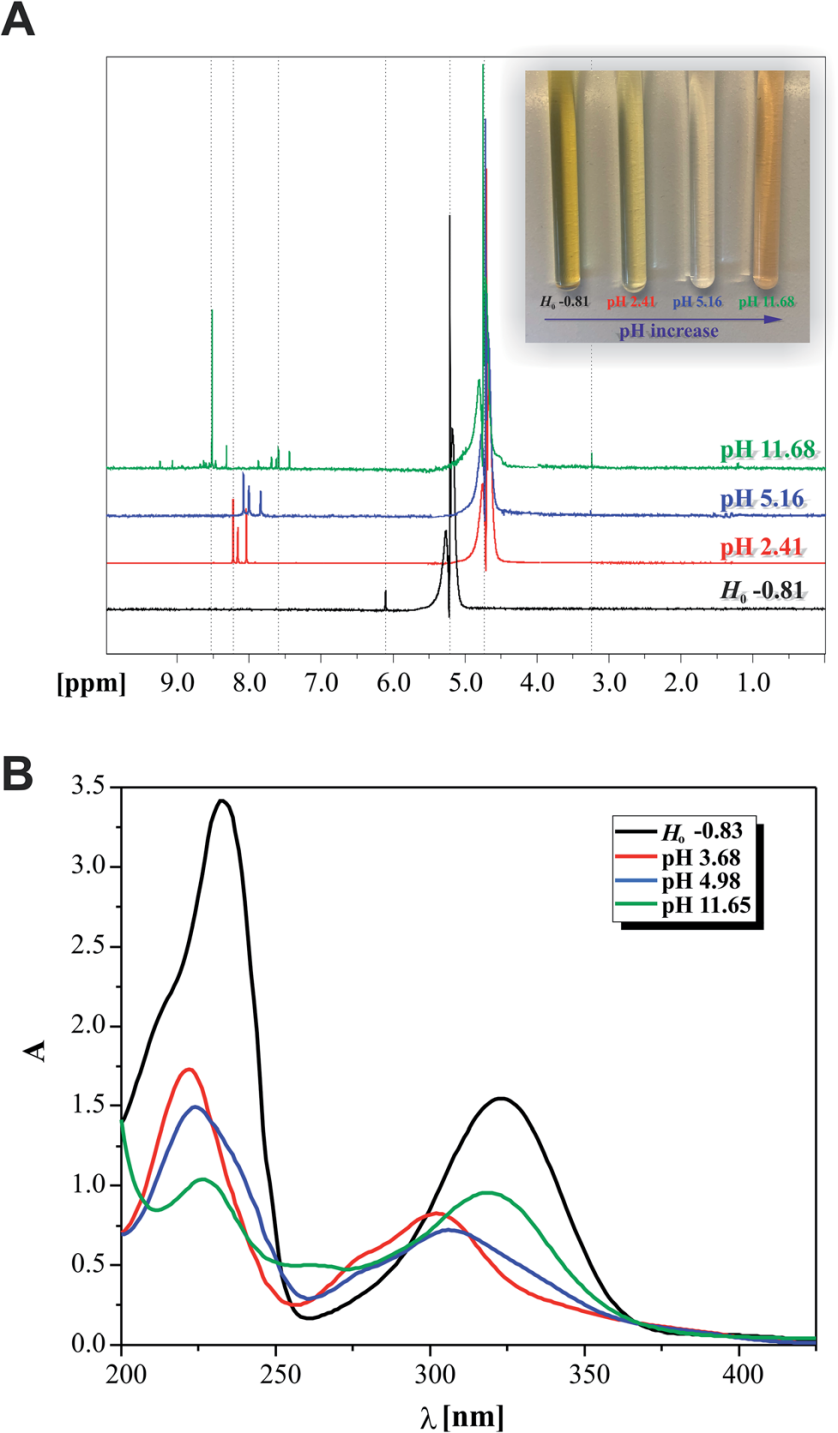
(A) NMR analysis of the four 2HP ionic forms spectra recorded at selected *H*_o_/pH values for the media studied: −0.81; 2.41; 5.16 and 11.68 (298 K); 2HP solutions with different pH values in NMR cuvettes (inset photograph). (B) 2HP UV spectra selected for the comparison analysis.

To resolve some controversy related with the 2HP ionic forms created during the protonation and deprotonation processes studied, the NMR protonic spectra of the four 2HP selected probes at special values of *H*_o_ and pH were also recorded and analyzed. This NMR investigation was performed intentionally to identify the 2HP parts that changed due to the presence of strong perchloric acid and strong potassium hydroxide (excluding the measured sodium error of the potentiometric combination glass electrode). Indeed, the spectral data clearly shows that protonation reactions occurred as a result of the strong and concentrated (70%) HClO_4_ (black spectrum in Fig. 9), which are directly related to the N heterocyclic atoms of the pyrazine ring of the 2HP structure. The NMR data also showed that this is due to the 2HP deprotonation species formed in the aqueous medium, as also shown in the previous UV spectroscopy and potentiometry experiments. The presence of the neutral and monoanionic forms of 2HP was proven by the shifts in the position of the NMR signals, as assigned in Fig. 9 (differences marked between red and blue spectra and between blue and green NMR spectra of 2HP samples). The regions of the shift changes indicated in Fig. 9A also prove that the 2HP ionization states of the described acid–base equilibria in the aqueous system (2.41 < pH < 11.68) refer strictly to the hydrazine substituent of 2HP. Moreover, the *σ* [ppm] signal shifts are in good agreement with the results for 2HP obtained through UV spectroscopy (photo on the right in Fig. 9A) and the discussion presented above. A comparison of the data obtained by the two independent experimental methods is presented in Fig. 9A and B. The electronic spectra of the 2HP ionic forms were selected among almost 158 recorded spectra and marked by corresponding colors to that presented for the NMR spectra.

### Evaluation of 2HP partition coefficient (log *P*)

An important aspect in physicochemical characterization is determining lipophilicity, which refers to the ability of a compound to dissolve in nonpolar systems. It is an indicator that allows the behavior of compound to be predicted in different media such as body fluids. Usually, lipophilicity is expressed as the partition coefficient. This value can be determined by experimental methods such as the shake-flask or pH-metric methods, but also by computational chemistry methods. The theoretical determination of log *P* is based on the following equation:^23,40,64^5
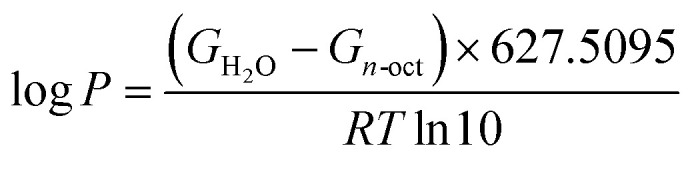


Here, the log *P* value was determined using the a standard system *n*-octanol/water, and the calculated values are presented in Table 3. All the values are negative, which clearly indicates the hydrophilic character of 2HP.

Subsequently, the experimental part of this assay was based on the UV light absorption for aqueous and octanolic samples of 2HP. These studies were specially designed using the optimal conditions and performed by recording the electronic spectra of the initial 2HP patterns in water and nonaqueous media and that of the individual extraction phases. Indeed, the 2HP patterns were used intentionally to estimate the real concentration of certain 2HP contaminants in both media. The spectrophotometric assay for the determination of the hydrophilic nature of 2HP included, in the first step, obtaining the proper 2HP concentration calibrations in both water and octanol (Fig. S1–S5, ESI).† Based on the calibration formulas, the proper 2HP concentration values were calculated as a result of the extractions. The electronic spectrum of each 2HP fraction was recorded (Fig. S6 of ESI†). All extraction measurements were repeated five times for a comparison of the results and to evaluate the repeatability of the experiments. The individual values of the equilibrium concentrations (*C*′) are presented in Table 4. Additionally, the 2HP distribution coefficients were determined as a percentage of the contaminants in the OCT/W systems and presented in Table 5. According to the experimental results and additional calculations, the log *P* value of −0.468 was obtained, which means that 2HP is hydrophilic in nature. This hydrophilic nature of 2HP is often preferred in the case of pharmacophore fragments substituted in the main skeleton of lipophilic pharmaceuticals in the drug design step. Moreover, 2HP does not form any aggregates or associates in an aqueous environment, as proven in Fig. S7 in the ESI.†

Results obtained (A) with theoretical methods: Gibbs free energy (*G*) in water and *n*-octanol and partition coefficient value (log *P*) and (B) with spectroscopy method: absorbance (*A*), concentration (*C*) in *n*-octanol (OCT) at 241 nm and water (W) at 237 nm, partition (*P*) and partition coefficient (log *P*) based on the ratio of the concentrations (*C*_OCT_/*C*_W_) and difference in the logarithmic concentrations (log *C*_OCT_ − log *C*_W_)

**Table:** 

(A) Theoretical part			
Method	*G*_H_2_O_ [hartree per part]	*G*_*n*-octanol_ [hartree per part]	log *P*
SMD/M06-2X/6-311+G**	−374.886949	−374.884054	−1.330
PCM/B3LYP/6-311+G**	−375.673558	−375.072235	−0.609
SMD/B3LYP/6-311+G**	−375.045634	−375.042571	−1.409

**Table:** 

(B) Experimental part								
OCT (241 nm)			W (237 nm)			*P*	log *P*	log *P*
*A*	*C* [M] ×10^−5^	log *C*	*A*	*C* [M] ×10^−5^	log *C*		*C*_OCT_/*C*_W_	log *C*_OCT_ − log *C*_W_
0.240	2.29	−4.640	0.824	7.16	−4.145	0.320	−0.49468	−0.49468
0.220	2.17	−4.663	0.808	7.01	−4.154	0.309	−0.50961	−0.50961
0.268	2.46	−4.609	0.807	7.01	−4.155	0.351	−0.45441	−0.45441
0.248	2.34	−4.631	0.817	7.09	−4.149	0.330	−0.48180	−0.48180
0.317	2.76	−4.560	0.800	6.94	−4.158	0.397	−0.40106	−0.40106
							log *P*	−0.468

**Table:** The amount of 2HP [%] in both OCT/W phases obtained as a result of extraction at two wavelengths (241 and 320 nm)

2HP content [%]			
OCT (240 nm)	OCT (320 nm)	W (240 nm)	W (320 nm)
37	35	63	65
37	37	63	63
36	34	64	66
37	34	63	66

The values obtained both experimentally and theoretically do not match quantitatively, but they all range from −0.5 to −1.5. As in the case of the p*K*_a_ constants, the deviation of the results obtained by theoretical methods arises from the use of approximations depending on the chosen method, functional base or solvent model.

### 2HP intermolecular interaction affinity with biomolecules

**Fig. 10:**
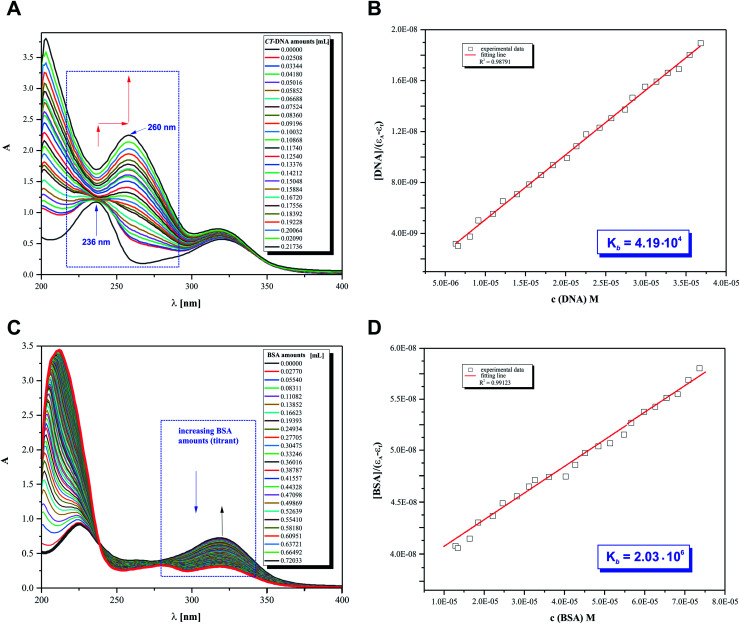
Absorption spectra of 2HP in Tris–HCl buffer upon the addition of (A) *CT*-DNA (0.37 mM) with subtraction of the *CT*-DNA absorbance ([2HP] = 0.18 mM). (B) Plot of [DNA]/(*ε*_a_ − *ε*_f_) *vs.* [DNA]. (C) BSA (0.25 mM) with subtraction of the BSA absorbance ([2HP] = 0.12 mM). (D) Plot of [BSA]/(*ε*_a_ − *ε*_f_) *vs.* [BSA]. The arrows show the absorbance changes associated with an increase in biomolecule concentration.

The main objective of our studies was to prove that 2HP is a low molecular, hydrophilic and biologically active molecule. Accordingly, an *in vitro* investigation was designed to allow the determination of the 2HP interaction modes with standard intracellular (bio)targets, namely *calf thymus*-DNA (*CT*-DNA) and bovine serum albumin (BSA). This was carried out *via* UV spectroscopy, which was completely automatized to keep the time between the addition of the biomolecule titrant constant and the same stirring time and titrant volume and ensured by computer control (Fig. 10A).

The UV spectra from the titrations of 2HP with DNA/BSA were examined at a constant pH value using the biological Tris–HCl buffer (pH 7.39) as a solvent for the preparation of each pattern solution sample. It can be observed that UV spectrum of 2HP exhibited two absorption bands in the absence of *CT*-DNA and BSA in the wavelength range of 200–400 nm. However, when the nucleic acid was added to the compound studied, significant changes in its UV spectrum occurred. The band intensity at 236 nm decreased and the peak maximum at 322 nm was remarkably enhanced with a red shift together with a hyperchromic effect observed. The presented spectral changes confirmed that interactions between 2HP and *CT*-DNA occurred in the system studied. Interestingly, although the second experiment with the BSA biomolecule was initially similar to the previous experiment, 2HP interacted with the protein with a definitely higher strength. Here, an isosbestic point appeared at 238 nm (Fig. 10C) and the peak maximum at 322 nm was again remarkably enhanced. According to the literature, the intensity of a band depends on the size of molecules and degree of electronic coupling among chromophores.^65^ The presence of an isosbestic point proved that at least one equilibrium occurring in the system studied is related to the formation of a 2HP–BSA adduct.

An adequate binding constant value (*K*_b_) for the quantitative comparison of the ability and binding strength of 2HP with *CT*-DNA and BSA was calculated based on the spectrophotometric titration data. The *K*_b_ value was determined based on the changes in absorbance of the π → π* peak after the addition of different amounts of individual biomolecule solution according to the presented Wolfe–Shimer equation (6):^66^6
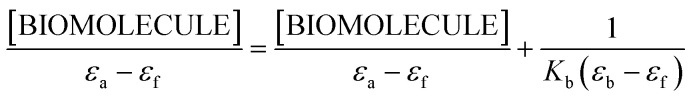
which includes the appropriate extinction coefficients (*ε*_a_ = *A*_obs_/[2HP]), where *ε*_f_ is determined using the spectrum of a free pyrazine derivative with a known concentration and *ε*_b_ established for 2HP-biomolecule adducts in the fully biomolecule bound form.

The dependence of [BIOMOLECULE]/(*ε*_a_ − *ε*_f_) as a function of [BIOMOLECULE] was plotted to determine the binding constant values (Fig. 10).

Interestingly, 2HP interacts with both biomolecules selected, *CT*-DNA and BSA. However, 2HP certainly shows higher affinity to albumin BSA than DNA. The binding constant values obtained suggest that BSA may act as a cellular target for the active 2HP molecule. The obtained values of *K*_b_ were estimated to be 4.19 × 10^4^ M^−1^ and 2.03 × 10^6^ M^−1^ for the formation of the 2HP–DNA and 2HP–BSA adducts, respectively. Based on the data, it can be concluded that the 2HP–BSA adducts created in the aqueous medium have higher stability compared to the 2HP–*CT*-DNA adducts.

### Cytotoxic activity

**Fig. 11:**
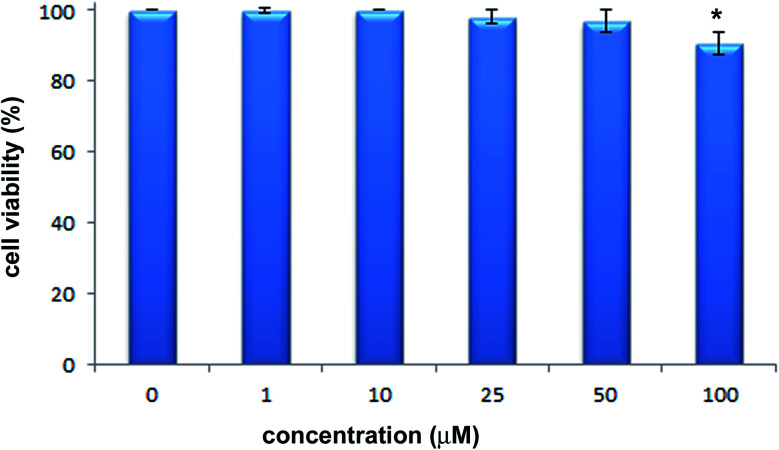
Effects of 2-hydrazinopyrazine toward HaCaT cells. Cell viability was analyzed with the use of the MTT assay after 72 h of incubation. Values represent mean ± SE of three independent experiments. *p* < 0.05 (*) indicates differences between the control and treated cells.

The cytotoxic activity of 2-hydrazinopyrazine was examined toward human keratinocytes, HaCaT cells. The effects of 2-hydrazinopyrazine toward HaCaT cells were determined using the MTT assay. This assay determines the metabolic activity of cells by analyzing the mitochondrial dehydrogenase conversion of tetrazolium salt to formazan. 2-Hydrazinopyrazine was examined in the concentration range of 1–100 μM, and its cytotoxicity was determined after 72 h of incubation with HaCaT cells. The results of the MTT assay revealed that 2-hydrazinopyrazine did not display cytotoxic activity in the examined concentration range. The viability of HaCaT cells was around 100% in the concentration range of 1–50 μM of the examined compound. At the highest tested concentration of 2-hydrazinopyrazine, 100 μM, the viability of HaCaT cells was determined to be ∼90% (Fig. 11).

## Conclusions

The physicochemical properties of 2HP were investigated by studying its structure, tautomerism, conformational isomerism, acid–base properties, and lipophilicity. Interestingly, four conformers were found upon searching the conformational hypersurface. The possibility of a transition between conformers was analyzed, together with the determination of the energy barriers for this transition. A total of 42 tautomeric forms were found, among which 12 are prototrophic forms. Three of them have relative Gibbs energies of less than 15 kcal mol^−1^, and therefore can exist and participate in the structure. The acid–base property assay revealed three possible 2HP protonation sites and one deprotonation site. A model of the equilibrium occurring in aqueous solution was proposed based on the energy analysis and potentiometric data. Consequently, the p*K*_a_ constant values of 2HP were determined. The hydrophilic character of 2HP was indicated by its negative log *P* values. The strong affinity of 2HP to interact with BSA was determined from the spectroscopic measurements. The strong 2HP–BSA binding mode was also confirmed by the higher binding constant obtained (*K*_b_ = 2.03 × 10^6^) for the 2HP–BSA system studied compared to that established for 2HP–DNA (*K*_b_ = 4.19 × 10^4^). Finally, the nontoxic nature of 2-hydrazinopyrazine was confirmed by the cytotoxicity assay.

In summary, 2-hydrazinopyrazine is a hydrophilic compound with no cytotoxic properties. 2HP interacts with different strengths with biomolecules such as BSA and DNA. Thus, the obtained profile of 2HP makes it attractive for further investigation.

## Conflicts of interest

There are no conflicts to declare.

## Supplementary Material

RA-010-D0RA06239A-s001
