# Supplementary material for: When biomolecules meet 2-hydrazinopyrazine: from theory through experiment to molecular levels using a wide spectrum of techniques
Source: RSC Adv. 2020 Nov 9;10(67):40673–88. doi: 10.1039/d0ra06239a (PMC9059145; doi:10.1039/d0ra06239a)
Supplement: RA-010-D0RA06239A-s001 [file RA-010-D0RA06239A-s001.pdf]

Electronic Supplementary Material (ESI) for RSC Advances.
This journal is © The Royal Society of Chemistry 2020

Supplementary Information

When biomolecules meet 2-hydrazinopyrazine: from theory, through experiment to molecular levels using wide spectrum of techniques

Paulina Mech[1], Mariusz Makowski[1], Anna Kawiak[2], Agnieszka Chylewska[1]

[1]University of Gdańsk, Faculty of Chemistry, Department of Bioinorganic Chemistry, ul. Wita Stwosza 63, 80-308 Gdańsk, Poland

[2]Intercollegiate Faculty of Biotechnology of University of Gdańsk and Medical University of Gdańsk, Laboratory of Plant Protection and Biotechnology, ul. Abrahama 58, 80-307 Gdańsk, Poland

*Corresponding author: agnieszka.chylewska@ug.edu.pl

Structure

Table S1. Bond lengths, angles and dihedral angles in optimized structure of 2-hydrazinopyrazine. Atom numbering shown on Fig. 1 (in main text). (Gibbs Free Energy: -375,028923 hartree/part.)

Atoms	Bond length [Å]	Atoms	Angle [°]	Atoms	Dihedral angle [°]
N1-C2	1.333	N1-C2-C3	121.051	N1-C2-N7-N8	-12.097
C2-C3	1.418	C2-C3-N4	121.753	N1-C2-N7-H12	-160.045
C3-N4	1.319	C3-N4-C5	117.183	C2-N7-N8-N13	79.692
N4-C5	1.346	N4-C5-C6	120.994	C2-N7-N8-H14	-35.500
C5-C6	1.385	C5-C6-N1	122.568		
C6-N1	1.341	C6-N1-C2	116.447		
C2-N7	1.379	N1-C2-N7	118.551		
N7-N8	1.408	C2-N7-N8	122.050		
C3-H9	1.088	C2-N7-H12	117.377		
C5-H10	1.084	N7-N8-H13	108.154		
C6-H11	1.086	N7-N8-H14	109.022		
N7-H12	1.008				
N8-H13	1.016				
N8-H14	1.019				

Table S2. Distribution of partial charges in neutral 2HP molecule determined using the NBO method on B3LYP/6-311+G** level of theory

Atom	Charge
N1	-0.58976
C2	0.51133
C3	-0.07654
N4	-0.44298
C5	-0.19146
C6	-0.02805
N7	-0.45311
N8	-0.80414
H9	0.25545
H10	0.26366
H11	0.25647
H12	0.45004
H13	0.42484
H14	0.42424

Table S3. Conformers 2HP(A)-2HP(D) and prototrophic forms (PA-PI3) of 2-hydrazinopyrazine with relative Gibbs free energy (ΔG) in kcal/mol (in relation to A conformer)

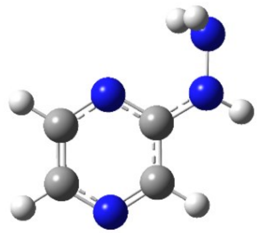	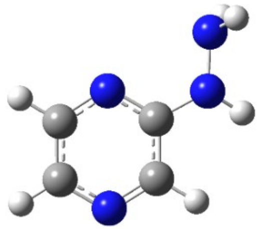	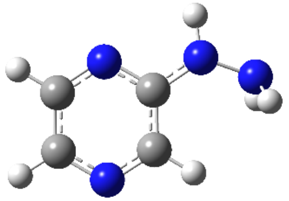	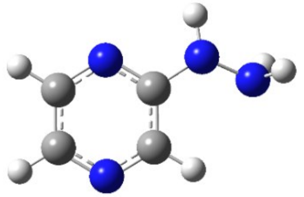		
**2HP(A)**<br>**ΔG: 0.00**	**2HP(B)**<br>**ΔG: 4.98**	**2HP(C)**<br>**ΔG: 1.16**	**2HP(D)**<br>**ΔG: 0.82**		
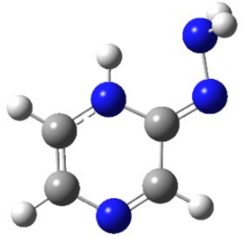	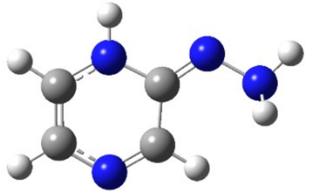	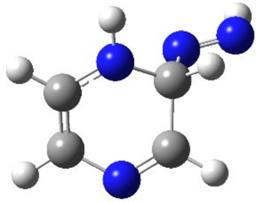	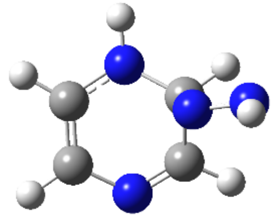	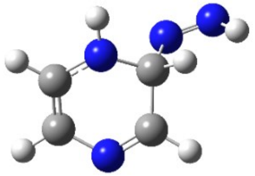	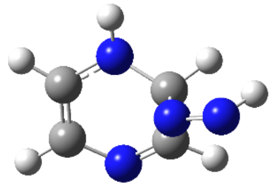
**PA**<br>**ΔG: 10.00**	**PA1**<br>**ΔG: 13.87**	**PA2**<br>**ΔG: 33.45**	**PA3**<br>**ΔG: 33.45**	**PA4**<br>**ΔG: 38.30**	**PA5**<br>**ΔG: 38.30**
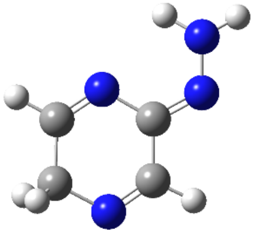	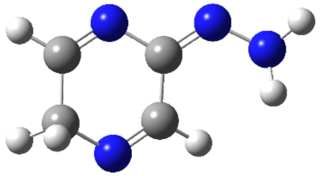	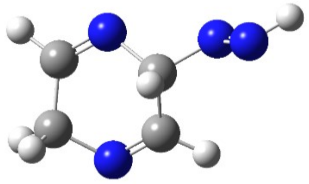	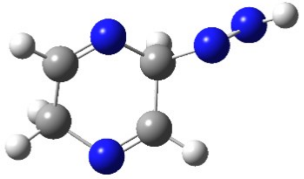	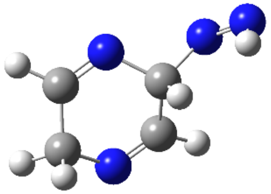	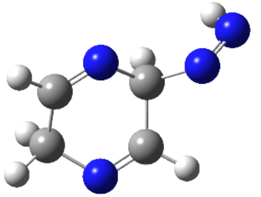
**PB**<br>**ΔG: 14.75**	**PB1**<br>**ΔG: 23.74**	**PB2**<br>**ΔG: 37.28**	**PB3**<br>**ΔG: 37.28**	**PB4**<br>**ΔG: 42.36**	**PB5**<br>**ΔG: 42.36**

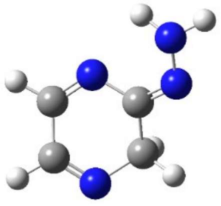	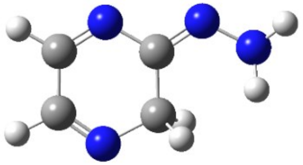	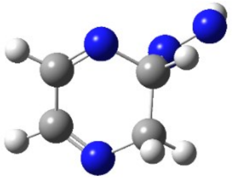	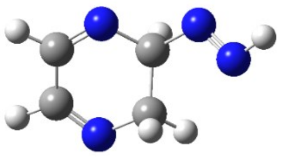	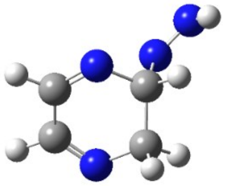	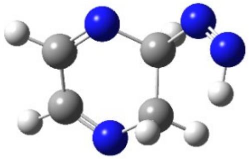
**PC**<br>**ΔG: 20.83**	**PC1**<br>**ΔG: 24.09**	**PC2**<br>**ΔG: 38.99**	**PC3**<br>**ΔG: 39.82**	**PC4**<br>**ΔG: 43.11**	**PC5**<br>**ΔG: 47.06**
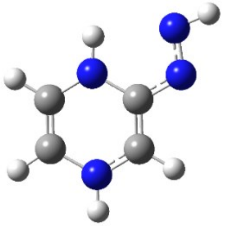	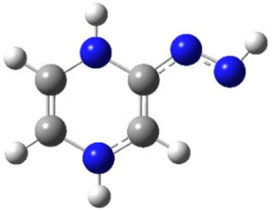	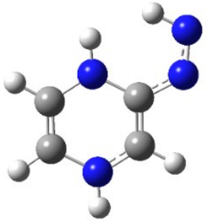	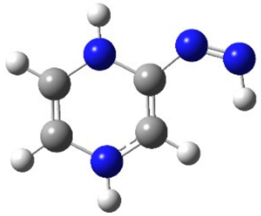		
**PD**<br>**ΔG: 27.82**	**PD1**<br>**ΔG: 37.02**	**PD2**<br>**ΔG: 37.60**	**PD3**<br>**ΔG: 44.47**		
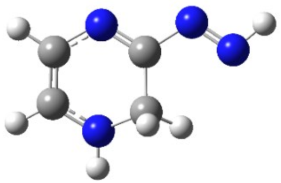	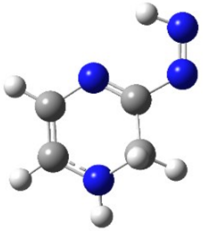	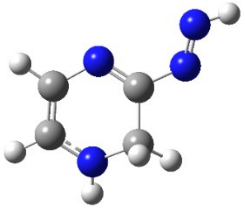	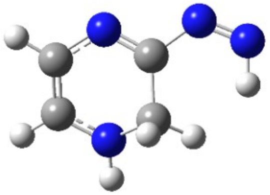		
**PE**<br>**ΔG: 28.48**	**PE1**<br>**ΔG: 32.11**	**PE2**<br>**ΔG: 32.16**	**PE3**<br>**ΔG: 37.13**		
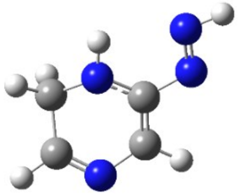	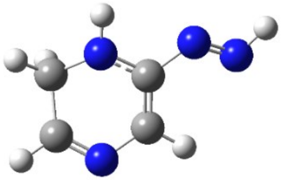	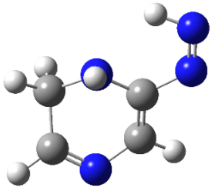	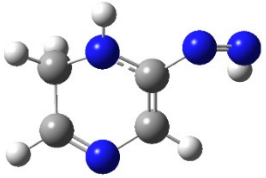		
**PF**<br>**ΔG: 28.74**	**PF1**<br>**ΔG: 33.89**	**PF2**<br>**ΔG: 36.55**	**PF3**<br>**ΔG: 38.63**		

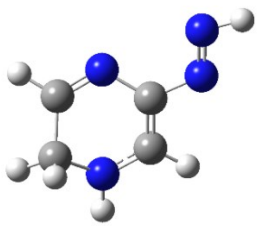	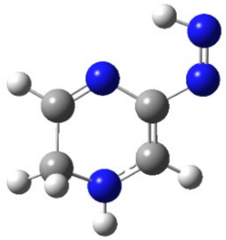	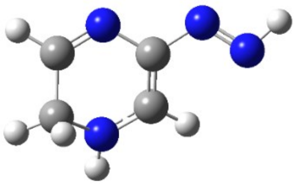	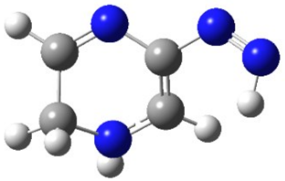
**PG**<br>**ΔG: 30.12**	**PG1**<br>**ΔG: 30.31**	**PG2**<br>**ΔG: 30.77**	**PG3**<br>**ΔG: 36.78**
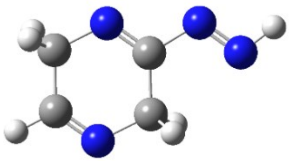	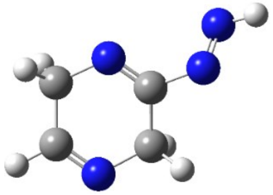	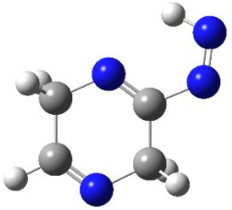	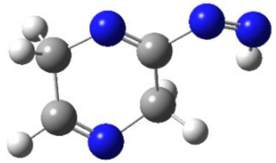
**PH**<br>**ΔG: 33.59**	**PH1**<br>**ΔG: 33.83**	**PH2**<br>**ΔG: 34.37**	**PH3**<br>**ΔG: 40.72**
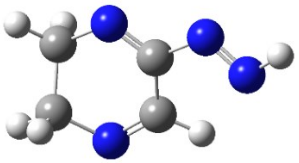	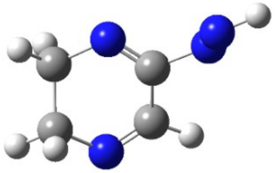	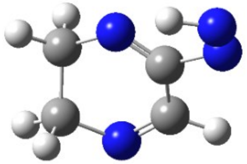	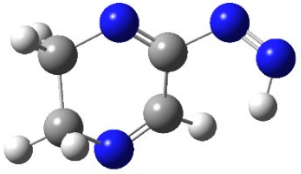
**PI**<br>**ΔG: 35.89**	**PI1**<br>**ΔG: 36.94**	**PI2**<br>**ΔG: 37.62**	**PI3**<br>**ΔG: 42.96**

Proton transfer process

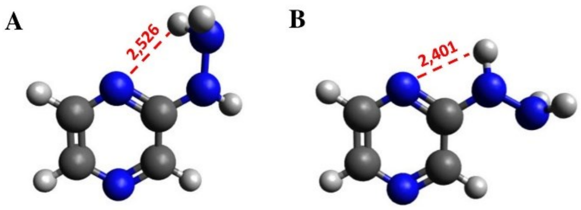

Figure S1. Structure of **A.** 2HP(A) and **B.** 2HP(D) conformers with marked hydrogen bonds and their lengths [Å]

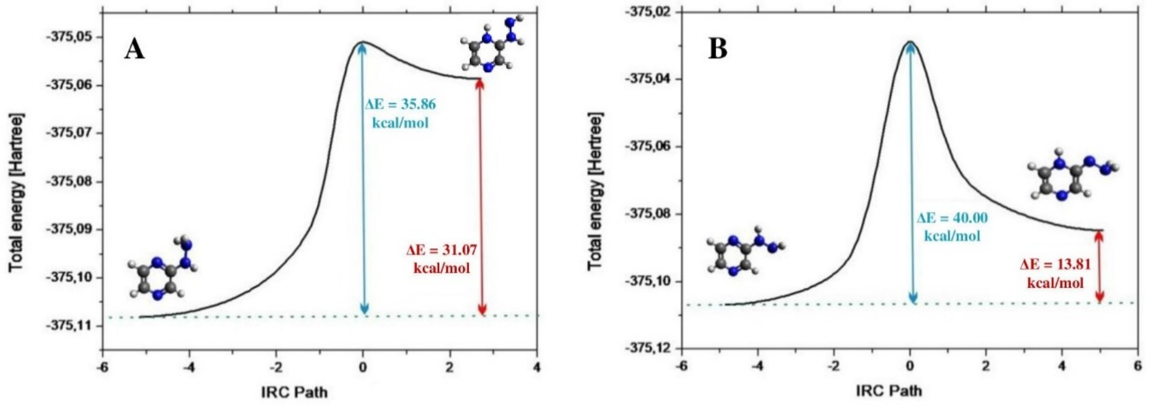

Figure S2. Total energy variation curve on IRC Path length during IRC proton transfer process analysis in **A.** 2HP(D) and **B.** 2HP(A). Energy change values have been converted to kcal/mol with conversion factor 627.5095

2HP partition (P) coefficient

The curves relate the measured absorbance by the UV spectrophotometer to the actual concentration of the 2HP in aqueous solution were registered (Figure S3).

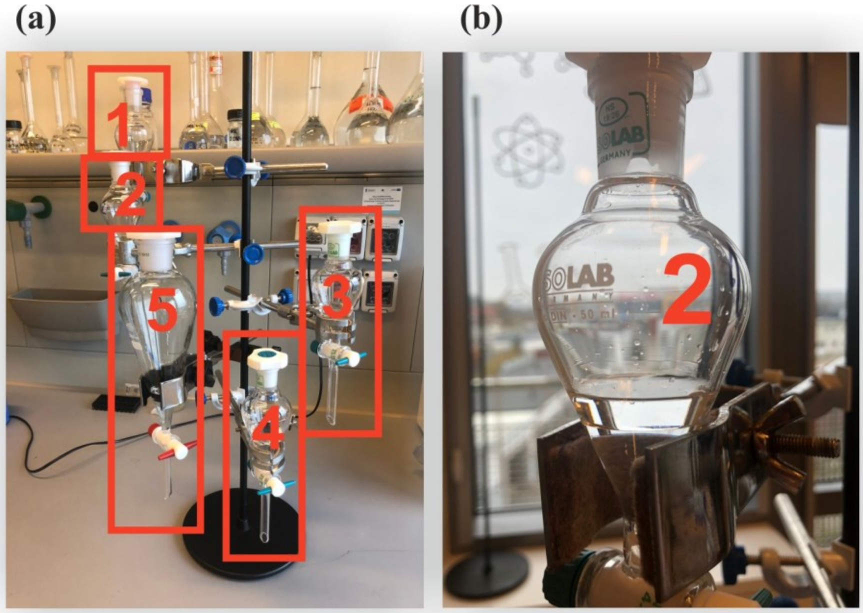

Figure S3. Spectrophotometrical experiment of the *2HP* logP determination: **(a)** five samples before the extraction **(b)** the second solution after 45 minutes of extraction.

Based on the initial condition it is obvious that the *2HP* octanolic solution absorbs UV radiation at 241 and 327 nm, but the absorption process for *2HP* in aqueous solvent can be observe at 237 and 321 nm of wavelengths. Both calibration curves for aqueous and octanolic samples as well as the confirmation of the Lambert-Beer's law of absorption are correlated, see *Figure S4*. The linear relationships between absorbance values and concentrations are very high and their values were presented in *Figure S5*. The *2HP* aqueous concentrations (C) is related to absorbance (A) by the equations inside the adequate calibration equations inside the *Figure S5(a) and Figure S5(b).* These expressions mentioned were also used to calculate the unknown concentration of *2HP* in aqueous and octanol fractions received as a result of extractions in the O/W system studied (*Figure S6*).

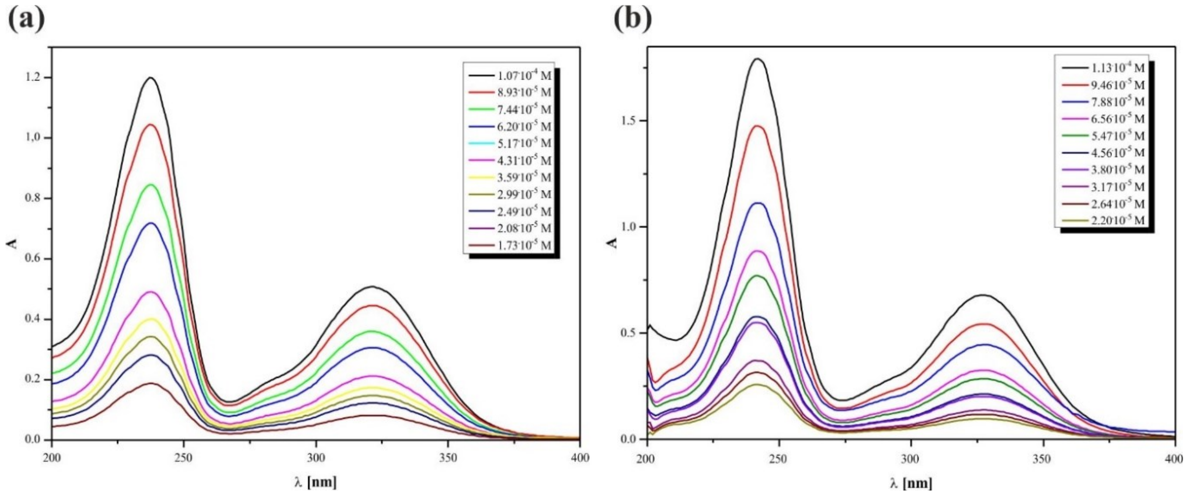

Figure S4. Lambert-Beer law as spectra of *2HP* in: **(a)** aqueous and **(b)** octanol solutions.

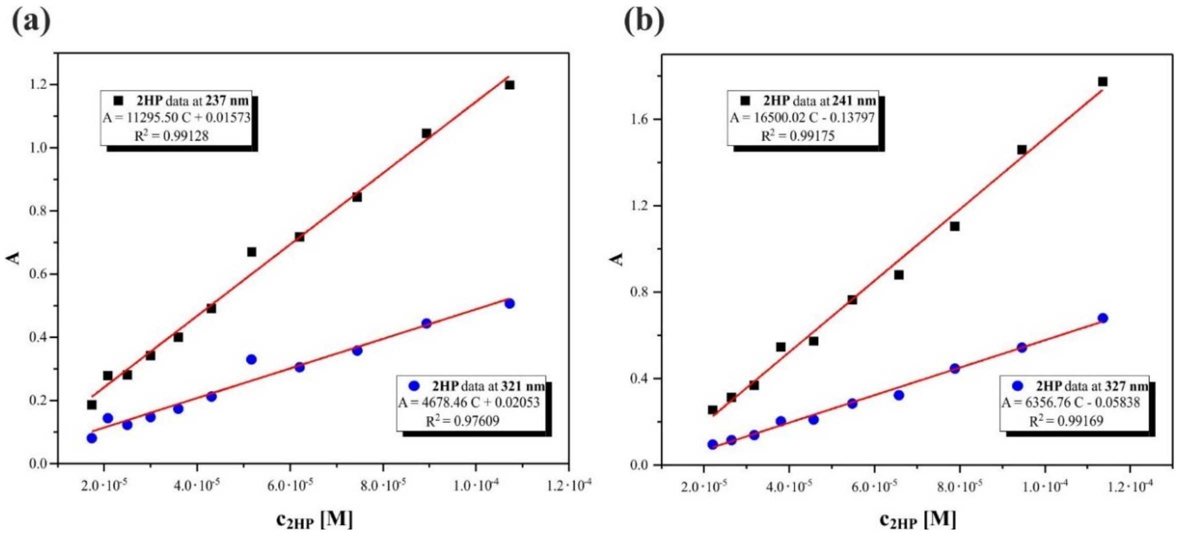

Figure S5. The relationships between absorbance and concentration of *2HP* in: **(a)** aqueous and **(b)** octanol solutions.

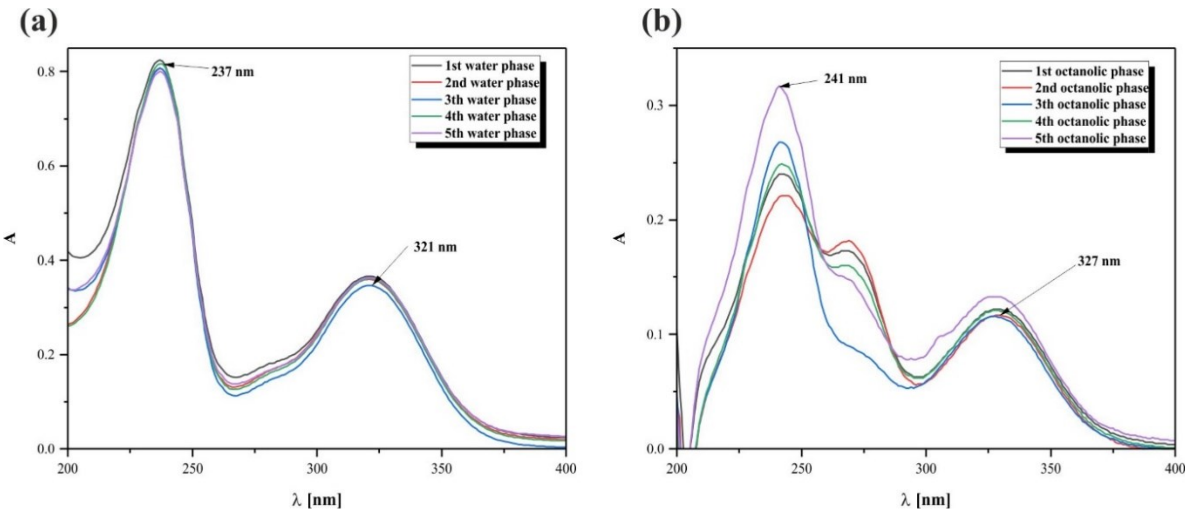

Figure S6. Electronic spectra obtained for aqueous **(a)** and octanol **(b)** fractions as a result of extractions for the initial *2HP* solution (O/W system studied).

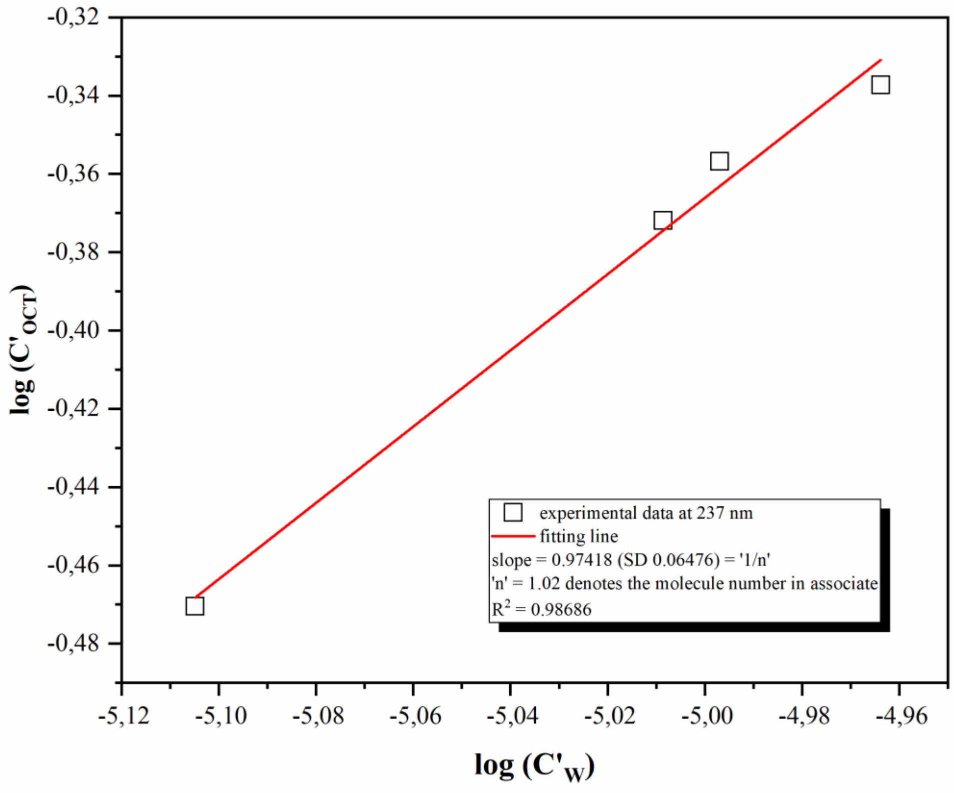

Figure S7. The graphical representation of ‘n’ parameter determination method (O/W system studied) to define the number of *2HP* molecules associated at 237 nm.
